# [^18^F]SMBT‐1 PET as an Imaging Biomarker for Reactive Astrogliosis in Mild Cognitive Impairment and Alzheimer's Disease

**DOI:** 10.1002/alz70856_107619

**Published:** 2026-01-09

**Authors:** Nobuyuki Okamura, Aiko Ishiki, Kotaro Hiraoka, Ryota Kobayashi, Naoki Tomita, Berihu Mesfin, Yingying Wu, Ryuichi Harada, Asuka Kikuchi, Kazuko Takeda, Akio Kikuchi, Katsutoshi Furukawa, Hiroshi Watabe, Shunji Mugikura, Shinobu Kawakatsu, Kenji Ishii, Takashi Nihashi, Takashi Kato, Shozo Furumoto, Manabu Tashiro

**Affiliations:** ^1^ Tohoku Medical and Pharmaceutical University, Sendai, Miyagi, Japan; ^2^ Research Center for Accelerator and Radioisotope Science, Tohoku University, Sendai, Miyagi, Japan; ^3^ Yamagata University Hospital, Yamagata, Yamagata, Japan; ^4^ Tohoku University Hospital, Sendai, Miyagi, Japan; ^5^ Fukushima Medical University, Aizu‐Wakamatsu, Fukushima, Japan; ^6^ Tokyo Metropolitan Institute for Geriatrics and Gerontology, Tokyo, Tokyo, Japan; ^7^ National Center for Geriatrics and Gerontology, Obu, Aichi, Japan

## Abstract

**Background:**

[^18^F]SMBT‐1, a PET tracer targeting monoamine oxidase B (MAO‐B), was developed to visualize reactive astrogliosis in the brain. This study investigated [^18^F]SMBT‐1 binding in the brains of patients with Alzheimer's disease (AD) and its association with plasma biomarkers.

**Method:**

We performed [^18^F]SMBT‐1 scans in 35 healthy elderly controls (HCs), 44 patients with mild cognitive impairment (MCI), and 13 patients with Alzheimer's disease (AD). To compare the regional standardized uptake value ratio (SUVR) between the disease groups, 30‐minute dynamic scans were conducted 60 min after administration of [^18^F]SMBT‐1. PiB PET or flutemetamol PET was performed to confirm the presence of amyloid‐β (Aβ) pathology in the brain. Plasma biomarkers (GFAP, pTau‐217, NfL) were measured in 38 subjects who underwent SMBT‐1 PET.

**Result:**

Compared to the amyloid‐negative HC and MCI groups, [^18^F]SMBT‐1 accumulation was significantly increased in many brain regions, including the temporal, parietal, occipital, cingulate, and parahippocampal gyri, in amyloid‐positive MCI and AD patients. These brain regions coincided with regions considered to be frequent sites of reactive astrogliosis in the AD continuum and overlapped with Aβ accumulation. Plasma GFAP was elevated in MCI and AD patients who showed the elevation of [^18^F]SMBT‐1 binding in the neocortex.

**Conclusion:**

[^18^F]SMBT‐1 binding in the neocortex was elevated in patients with MCI and AD in association with Aβ accumulation, which is consistent with the results of plasma GFAP measurements. These findings suggest that [^18^F]SMBT‐1 PET is a useful biomarker of reactive astrogliosis in the brain.

